# Acute Congestion of the Lumbar Region of the Spinal Cord, Cystitis, Pyelitis, Acute Parenchymatous Nephritis—Posterior Paralysis of the Horse

**Published:** 1881-07

**Authors:** George H. Parkinson

**Affiliations:** Middletown, Conn.


### IV.—ACUTE CONGESTION OF THE LUMBAR REGION OF THE SPINAL CORD, CYSTITIS, PYELITIS, ACUTE PARENCHYMATOUS NEPHRITIS. POSTERIOR PARALYSIS OF THE HORSE.

REPORTED BY GEORGE H. PARKINSON, D.V.S.

A gelding, aged 10 years, had always been in good health prior to the present attack. This animal was used for light farm work, and only occasionally driven on the road, and then at a slow pace. The cause of this attack is rather obscure, for the horse had not been exposed to wet or cold, nor does the attack seem to have supervened upon driving, although the first symptom of posterior weakness was noticed on the road. For several days before I attended the animal he was noticed to stumble, which difficulty increased, with a gradual loss of power in the posterior extremities. When I first saw the animal he was lying down, and was positively unable to get up. The pulse was beating from 45 to 46 per minute. Temperature, 102° F. Respiration quickened, with a spasmodic action of the diaphragm. The abdomen was distended with gas. There was almost complete loss of motion and sensation in the posterior extremities. The bladder was enormously distended with wind, rendering the passage of the hand into the rectum difficult and almost impossible. All the visible mucous mem branes were injected. The appetite was poor, accompanied by great thirst. The skin was neither moist nor dry, but nearly normal. The animal was somewhat restless, and, from the constant rubbing of the head upon the floor, the eyes became very much inflamed, otherwise they appeared normal. There was no swelling or tenderness that could be detected in the lumbar region.

At first there was an increased quantity of urine, of a light amber color; but, as the case progressed, the quantity steadily became less, and the color of a deeper hue. All power to void the urine was lost, and the catether had to be used. On the second day after I first saw the animal the temperature fell, and remained nearly normal up to the day of death.

The treatment consisted in emptying the bladder with a catheter, cathartics, and balls containing digitalis and nux vomica. At the end of seven days the animal had not improved in any respect, and he was forthwith ordered to be killed.

At the necropsy I found the following conditions: The lesions were located principally in the genito-urinary tract and in the spinal cord, the other organs of the body appearing normal. The mucous membrane of the bladder was congested, and showed evidence of acute and chronic inflammation, and at one point it was destroyed by an ulcerative process, leaving a raw surface the size of a half-dollar. The bladder was empty. The ureters and pelvis of the kidneys were deeply congested, and in the pelvis numerous ecclymotic spots were present. The pelvis of the kidneys also contained a little purulent fluid. The kidneys were hard and very deeply congested.

The microscopical examination of all the organs was made by Dr. Porter, at the School of Histology in connection with the Columbia Veterinary College, New York City, who found the kidneys to be in an advanced state of acute parenchymatous degeneration. The epithelium was finely granular and swollen, and in many of the tubules entirely wanting. Numerous blood corpuscles were present in each section; also pigment matter, probably blood pigment.

*Spinal Cord.*—There was no fluid in the subarachnoid space. The larger vessels of the meninges, covering the lumbar and sacral portion of the cord, were distended with blood, but there was not the slightest evidence of any inflammatory exudation. This congestion was also especially well marked on the spinal nerves at the point where they passed through the dura-mater, and probably produced considerable pressure on the nerves at this point. The cut surface of a tranverse section showed the blood-vessels as so many bright red spots and lines, the spots looking in some sections like infarctions. Microscopically, these bright red points, visible to the naked eye, proved in thin sections to be blood-vessels distended with blood corpuscles, and in some sections evidences of slight extravasation were present. The congestion was most marked in the anterior horns of the gray matter. Some of the vessels running from the surface of the cord to the gray nerve centres were distended throughout their whole course. The neuroglia interspaces were apparently distended, as if some oedema of the cord had existed during life.

MIDDLETOWN, CONN.

